# Planar *versus* non-planar: The important role of weak C—H⋯O hydrogen bonds in the crystal structure of 5-methyl­salicyl­aldehyde

**DOI:** 10.1107/S2056989017000238

**Published:** 2017-01-13

**Authors:** Ulrich Baisch, Marie Christine Scicluna, Christian Näther, Liana Vella-Zarb

**Affiliations:** a Department of Chemistry, University of Malta, Msida, MSD 2080, Malta; b Anorganische Chemie, Christian-Albrechts-Universität zu Kiel, Max-Eyth-Str 2, 24118 Kiel, Germany

**Keywords:** crystal structure, 5-MSA, organic, salicylic acid, hydrogen bonds

## Abstract

The crystal structure of 5-methyl­salicyl­aldehyde (**5-MSA**; systematic name 2-hy­droxy-5-methyl­benzaldehyde), C_8_H_8_O_2_, was discovered to be a textbook example of the drastic structural changes caused by just a few weak C—H⋯O inter­actions due to the additional methyl­ation of the aromatic ring compared to salicyl­aldehyde **SA**. This weak inter­molecular hydrogen bonding is observed between aromatic or methyl carbon donor atoms and hydroxyl or aldehyde acceptor oxygen atoms with *d*(*D*⋯*A*) = 3.4801 (18) and 3.499 (11) Å. The mol­ecule shows a distorted geometry of the aromatic ring with elongated bonds in the vicinity of substituted aldehyde and hydroxyl carbon atoms. The methyl hydrogen atoms are disordered over two sets of sites with occupancies of 0.69 (2) and 0.31 (2).

## Abstract 2

Wavy layers of mol­ecules are formed in the crystal structure of 5-methyl­salicyl­aldehyde due to weak C—H⋯O inter­actions between methyl groups and the aromatic ring system. Mol­ecules form columns in which the methyl groups are oriented in opposite directions layer-by-layer along cell axis *a.* In the mol­ecule, the hydroxyl substituent is bound intra­molecularly to the aldehyde group at the *ortho* position.

## Chemical context   

Salicyl­aldehydes form an important and widely used group of compounds in the pharmaceutical and agrochemical industry (Kirchner *et al.*, 2011 ▸). They have a functional role as metabolites in eukaryotic plants and as nematicides (Caboni *et al.*, 2013 ▸; Kim *et al.*, 2008 ▸). As part of a series of co-crystallization experiments in which the title compound was used as a coformer, single-crystals of 5-methyl­ated salicyl­aldehyde (**5-MSA**) were obtained and characterized by single-crystal X-ray diffraction. Its crystal structure is reported herein and compared to the unsubstituted form of salicyl­aldehyde (**SA)** [Kirchner *et al.* (2011 ▸); refcode YADJOE in the Cambridge Structural Database (Groom *et al.*, 2016 ▸)]. Even though **5-MSA** carries just one additional methyl group compared to the latter, a very large difference in melting point is observed. Whereas **SA** is a liquid at room temperature, **5-MSA** is a crystalline solid with a melting point of 328–330 K.
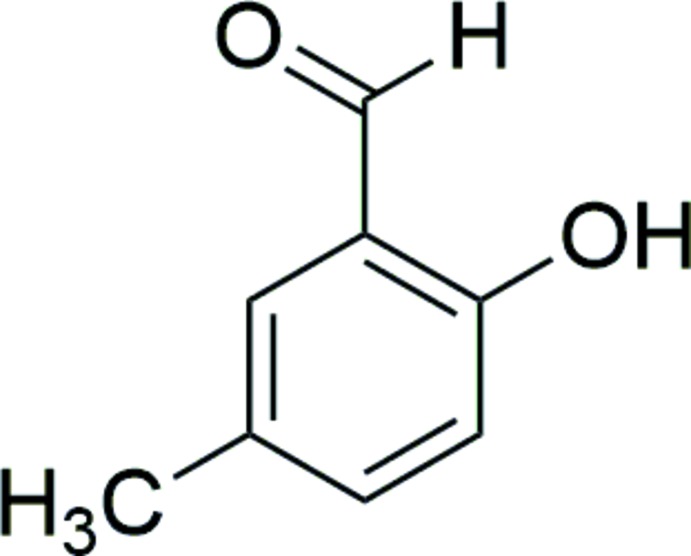


## Structural commentary   

**Figure 1:**
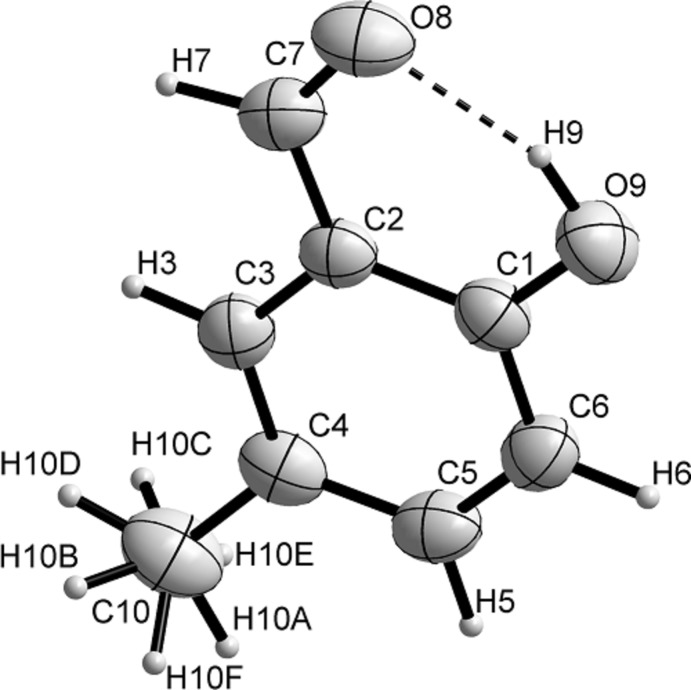
The mol­ecular structure of **5-MSA** showing the labeling scheme and anisotropic displacement ellipsoids drawn at the 50% probability level using *DIAMOND* (Brandenburg, 1999 ▸). The dashed line indicates the intra­molecular hydrogen bond.

The mol­ecular structure of **5-MSA** features a benzene ring (C1–C6), carrying a hydroxyl substituent at position 1, which is bound intra­molecularly to the aldehyde group at the *ortho* position by a fairly strong hydrogen-bond inter­action with *d*(D⋯*A*) = 2.6260 (17) Å (Fig. 1 ▸). In the aromatic ring, the adjacent hydroxyl and aldehyde groups, as well as the methyl­ated C4 atom, lead to a distortion of its geometry, expressed by the slight increase in the C1—C2, C2—C3 and C4—C5 bond lengths to 1.4028 (18) Å, 1.4001 (18) Å, and 1.398 (2) Å, respectively. The other bonds of the ring lie within the expected range, exhibiting the usual lengths of aromatic carbon–carbon bonds [C3—C4 = 1.3781 (19) Å, C5—C6 = 1.377 (2) Å and C1—C6 1.3879 (19) Å]. This affects the corresponding bond angle C3—C4—C5 in the ring, which is 117.16 (13)°. The distance of atom C2 from the aldehyde carbon atom C7 is 1.4507 (18) Å and the deviation from the mean plane defined by the aldehyde and the aromatic ring can be established by the torsion angles C7—C2—C3—C4 [−177.15 (12)°] and C7—C2—C1—C6 [176.64 (11)°]. A similar distortion is observed at torsion angles C7—C2—C1—O9 [−3.38 (18)°] and C1—C2—C7—O8 [2.7 (2)°]. This particular geometry may facilitate the intra­molecular O9—H9⋯O8 hydrogen bond [*d*(O9⋯O8) = 2.6260 (17) Å; O9—H9⋯O8 = 152 (2)°;Table 1 ▸]. In comparison, the corres­ponding hydrogen-bonding inter­action in **SA** has *d*(*D*⋯*A*) = 2.6231 (17) Å and an angle of 156°. The benzene ring in **5-MSA** also carries a methyl substituent at the 5-*meta* position, with a C4—C10 bond length of 1.505 (2) Å.

## Supra­molecular features   

**Figure 2:**
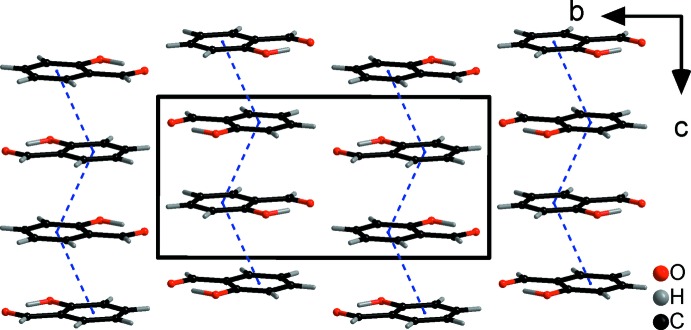
The crystal packing (*DIAMOND;* Brandenburg, 1999 ▸) of **SA** viewed along the *a* axis. π-stacking inter­actions are indicated by blue dashed lines.

The large difference in melting point between **SA** and **5-MSA** is unequivocally related to the different way the two mol­ecules pack in the crystal lattice. Layers of **SA** mol­ecules are arranged in almost perfect sheets, resulting in a layered structure roughly along the *a* axis. The distance between these layers of mol­ecules can be analysed by the distance between the centroids (*Cg*) of the phenyl rings with *d*(*Cg*⋯*Cg*) = 3.7838 (11) Å (Figs. 2 ▸ and 3 ▸). No inter­molecular hydrogen-bonding inter­actions can be detected in the range *d*(*D⋯A*) = 2.5–3.5 Å.

**Figure 4:**
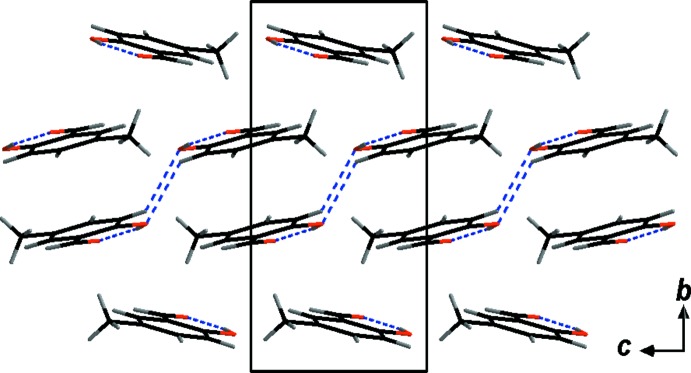
The crystal packing (*DIAMOND;* Brandenburg, 1999 ▸) of **5-MSA** viewed along the *a* axis. Hydrogen-bonding inter­actions are shown as blue dashed lines.

The **5-MSA** mol­ecules do show some inter­esting inter­molecular inter­actions (Steiner, 2002 ▸) in the same range [*d*(*D⋯A*) = 2.5–3.5 Å] apart from van der Waals inter­actions. Three C—H⋯O inter­actions are present between either aromatic or methyl C atoms and aldehyde or alcohol oxygen atoms: two close to 3.5 Å with C10⋯O8 = 3.499 (2) Å and C5⋯O8 = 3.4801 (18) Å and corresponding C—H⋯O angles of 152 and 149.3 (13)°, respectively. The third and shortest inter­action, has a C6⋯O9 distance of 3.4053 (18) Å and an angle of 138.7 (12)° (Table 1 ▸). The latter results in a 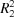
(8) ring, a graph set very often observed in the centrosymmetric structures of aromatic acids and aldehydes due to the occurrence of inversion centres between mol­ecules (Fig. 4 ▸). In this manner, pairs of mol­ecules are connected to each other by weak inter­molecular inter­actions.

**Figure 5:**
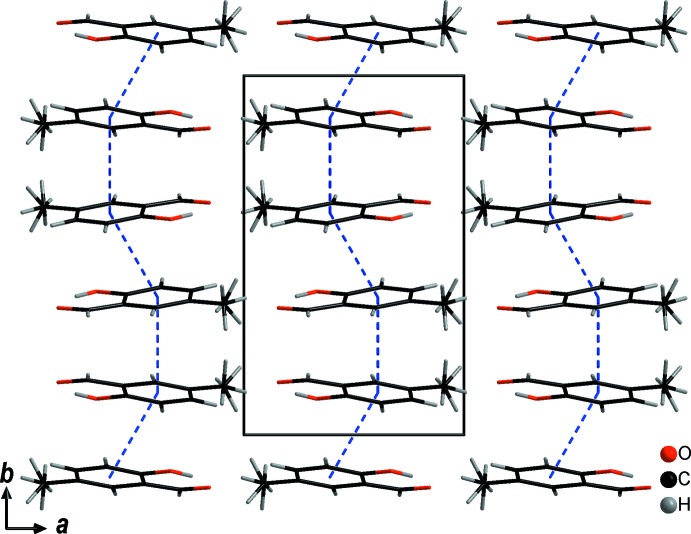
The crystal packing (*DIAMOND;* Brandenburg, 1999 ▸) of **5-MSA** viewed along the *c* axis. π-stacking inter­actions are indicated by blue dashed lines drawn between the centroids of the aromatic rings.

The most significant consequence of the additional inter­actions compared to **SA**, however, can be seen in the distances between the phenyl rings and the geometry of how they are arranged towards each other. There are two distances between the centroids of the phenyl rings, one within significance range, the other one slightly above, with *d*(*Cg*⋯*Cg*) = 3.7539 (11) and 4.7456 (13) Å, respectively. This results in a deviation from the usually expected herringbone or completely planar arrangement of planar mol­ecules. Wavy layers of mol­ecules are formed instead, whereby the **5-MSA** mol­ecules form columns in which the methyl groups are oriented in opposite directions layer-by-layer along the *a* axis (Figs. 5 ▸ and 6 ▸).

The stronger π-stacking of the aromatic rings combined with the additional weak inter­molecular inter­actions provides a logical explanation for the difference in melting points between **SA** and **5-MSA** and is a perfect textbook example of the drastic structural changes caused by just a few weak C—H⋯O inter­actions due to an additional methyl­ation of the aromatic ring.

## Synthesis and crystallization   

The title compound, together with a catalytic volume of ethanol solvent, was ground in a mortar and pestle into a dried powder, which was then dissolved in 1.5 mL of the solvent and allowed to crystallize. Single crystals of suitable quality were selected directly from the dried crystalline precipitate.

## Refinement   

Crystal data, data collection and structure refinement details are summarized in Table 2 ▸. The structure solution was not straightforward. A first attempt to solve the structure in space group *P*2_1_/*c* was unsuccessful. The structure solution was carried out in *P*1 and then transformation using *PLATON* (Spek, 2009 ▸) to the correct space group *P*2_1_/*c* took place. The hydrogen atoms of the methyl substituent show disorder with an occupancy of 0.69 (2) at positions H10*A*, H10*B*, H10*C* and 0.31 (2) at positions H10*D*, H10*E*, H10*F*. They were included at idealized positions riding on the parent carbon atom, with isotropic displacement parameters *U_i_*_so_(H) = 1.5*U*_eq_(CH_3_). Refinement of the corresponding site-occupation factors of the methyl-group hydrogen atoms was carried out using a free variable so that their sum is unity. All other hydrogen atoms were located individually in a difference-Fourier map and refined isotropically.

## Supplementary Material

Crystal structure: contains datablock(s) I. DOI: 10.1107/S2056989017000238/lh5831sup1.cif

Structure factors: contains datablock(s) I. DOI: 10.1107/S2056989017000238/lh5831Isup2.hkl

Supporting information file. DOI: 10.1107/S2056989017000238/lh5831Isup3.cml

CCDC reference: 1525796

Additional supporting information:  crystallographic information; 3D view; checkCIF report

## Figures and Tables

**Figure 3:**
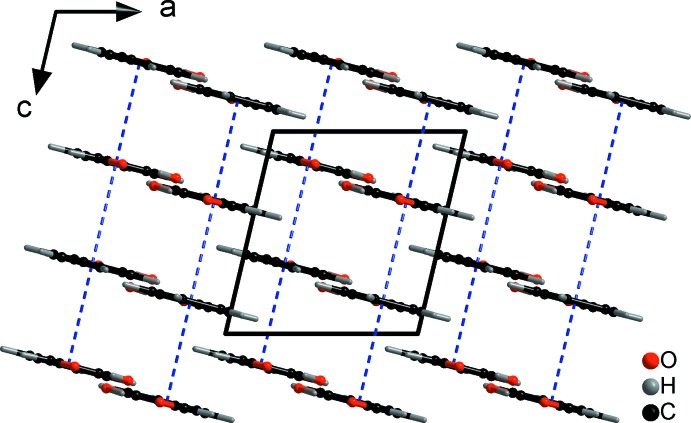
The crystal packing (*DIAMOND;* Brandenburg, 1999 ▸) of **SA** viewed along the *b* axis. π-stacking inter­actions are indicated by blue dashed lines.

**Figure 6:**
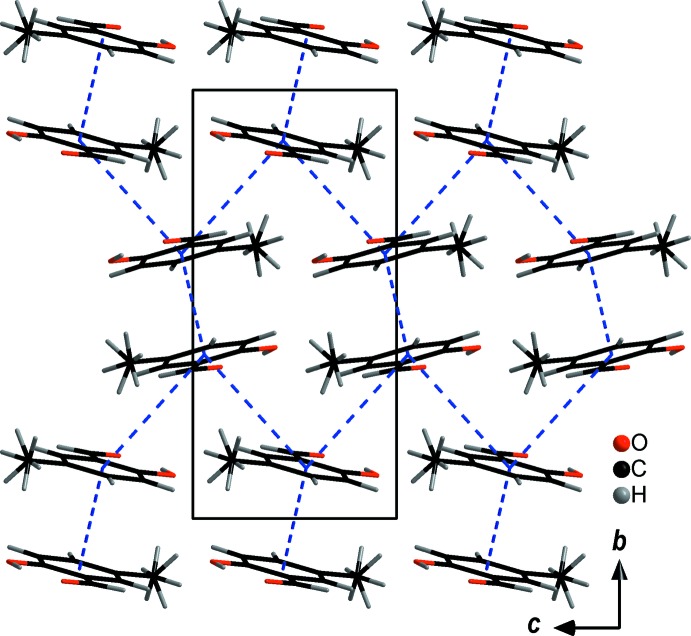
The crystal packing (*DIAMOND;* Brandenburg, 1999 ▸) of **5-MSA** viewed along the *a* axis. π-stacking inter­actions are indicated by blue dashed lines drawn between centroids of the aromatic ring.

**Table 1:** Hydrogen-bond geometry (Å, °)

*D*—H⋯*A*	*D*—H	H⋯*A*	*D*⋯*A*	*D*—H⋯*A*
C10—H10*E*⋯O8^i^	0.98	2.60	3.499 (2)	152
O9—H9⋯O8	0.94 (3)	1.77 (3)	2.6260 (17)	151 (2)
C5—H5⋯O8^ii^	0.974 (18)	2.607 (18)	3.4801 (18)	149.3 (13)
C6—H6⋯O9^iii^	0.989 (16)	2.599 (17)	3.4053 (18)	138.7 (12)

Symmetry codes: (i) 
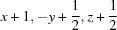
; (ii) 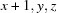; (iii) 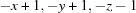.

**Table 2:** Experimental details

Crystal data	
Chemical formula	C_8_H_8_O_2_
*M*_r_	136.14
Crystal system, space group	Monoclinic, *P*2_1_/*c*
Temperature (K)	170
*a*, *b*, *c* (Å)	8.3676 (17), 13.088 (3), 6.4867 (13)
β (°)	106.30 (3)
*V* (Å^3^)	681.8 (3)
*Z*	4
Radiation type	Mo *K*α
μ (mm^−1^)	0.10
Crystal size (mm)	0.2 × 0.15 × 0.1

Data collection	
Diffractometer	STOE *IPDS2*
Absorption correction	–
No. of measured, independent and observed [*I* > 2σ(*I*)] reflections	7980, 1617, 1276
*R*_int_	0.049
(sin θ/λ)_max_ (Å^−1^)	0.658

Refinement	
*R*[*F* ^2^ > 2σ(*F* ^2^)], *wR*(*F* ^2^), *S*	0.045, 0.118, 1.07
No. of reflections	1617
No. of parameters	112
H-atom treatment	H atoms treated by a mixture of independent and constrained refinement
Δρ_max_, Δρ_min_ (e Å^−3^)	0.15, −0.11

Computer programs: *X-AREA* (Stoe & Cie, 2002 ▸), *SHELXS* (Sheldrick, 2008 ▸), *SHELXL* (Sheldrick, 2015 ▸), OLEX2 (Dolomanov *et al.*, 2009 ▸) and *DIAMOND* (Brandenburg, 1999 ▸).

## supplementary crystallographic information

### Crystal data

**Table:**

C_8_H_8_O_2_	*F*(000) = 288
*M**_r_* = 136.14	*D*_x_ = 1.326 Mg m^−^^3^
Monoclinic, *P*2_1_/*c*	Mo *K*α radiation, λ = 0.71073 Å
*a* = 8.3676 (17) Å	Cell parameters from 2429 reflections
*b* = 13.088 (3) Å	θ = 1.5–28.5°
*c* = 6.4867 (13) Å	µ = 0.10 mm^−^^1^
β = 106.30 (3)°	*T* = 170 K
*V* = 681.8 (3) Å^3^	Block, clear colourless
*Z* = 4	0.2 × 0.15 × 0.1 mm

### Data collection

**Table:**

STOE IPDS-2 diffractometer	*R*_int_ = 0.049
Graphite monochromator	θ_max_ = 27.9°, θ_min_ = 3.0°
ω scans	*h* = −10→10
7980 measured reflections	*k* = −17→17
1617 independent reflections	*l* = −8→8
1276 reflections with *I* > 2σ(*I*)	

### Refinement

**Table:**

Refinement on *F*^2^	0 restraints
Least-squares matrix: full	Hydrogen site location: mixed
*R*[*F*^2^ > 2σ(*F*^2^)] = 0.045	H atoms treated by a mixture of independent and constrained refinement
*wR*(*F*^2^) = 0.118	*w* = 1/[σ^2^(*F*_o_^2^) + (0.0511*P*)^2^ + 0.1028*P*] where *P* = (*F*_o_^2^ + 2*F*_c_^2^)/3
*S* = 1.07	(Δ/σ)_max_ < 0.001
1617 reflections	Δρ_max_ = 0.15 e Å^−^^3^
112 parameters	Δρ_min_ = −0.11 e Å^−^^3^

### Special details

**Table:**

Geometry. All esds (except the esd in the dihedral angle between two l.s. planes) are estimated using the full covariance matrix. The cell esds are taken into account individually in the estimation of esds in distances, angles and torsion angles; correlations between esds in cell parameters are only used when they are defined by crystal symmetry. An approximate (isotropic) treatment of cell esds is used for estimating esds involving l.s. planes.
Refinement. Chicken Wire Problem: first structure solution in P1 then transformation using Platon to P2(1)/c (Brandenburg, 1999)'

### Fractional atomic coordinates and isotropic or equivalent isotropic displacement parameters (Å^2^)

**Table:**

	*x*	*y*	*z*	*U*_iso_*/*U*_eq_	Occ. (<1)
O9	0.33041 (14)	0.39946 (8)	−0.40519 (16)	0.0531 (3)	
O8	0.15789 (12)	0.35357 (8)	−0.13645 (19)	0.0578 (3)	
C4	0.75281 (16)	0.37298 (10)	0.1197 (2)	0.0447 (3)	
C6	0.62263 (18)	0.41085 (10)	−0.2573 (2)	0.0462 (3)	
C2	0.45246 (15)	0.36322 (9)	−0.0290 (2)	0.0388 (3)	
C5	0.76193 (17)	0.40142 (10)	−0.0845 (2)	0.0469 (3)	
C10	0.90763 (19)	0.36471 (13)	0.3055 (3)	0.0618 (4)	
H10A	1.0054	0.3807	0.2564	0.074*	0.361 (18)
H10B	0.9008	0.4130	0.4182	0.074*	0.361 (18)
H10C	0.9175	0.2950	0.3630	0.074*	0.361 (18)
H10D	0.8771	0.3451	0.4353	0.074*	0.639 (18)
H10E	0.9817	0.3128	0.2735	0.074*	0.639 (18)
H10F	0.9649	0.4308	0.3288	0.074*	0.639 (18)
C3	0.59671 (17)	0.35444 (9)	0.1431 (2)	0.0419 (3)	
C7	0.29005 (17)	0.34759 (10)	0.0048 (2)	0.0471 (3)	
C1	0.46618 (16)	0.39116 (9)	−0.2321 (2)	0.0409 (3)	
H9	0.240 (3)	0.3860 (18)	−0.351 (4)	0.101 (8)*	
H3	0.5831 (18)	0.3354 (12)	0.278 (2)	0.046 (4)*	
H5	0.871 (2)	0.4169 (13)	−0.104 (3)	0.063 (5)*	
H6	0.630 (2)	0.4329 (12)	−0.400 (3)	0.057 (4)*	
H7	0.293 (2)	0.3328 (12)	0.158 (3)	0.054 (4)*	

### Atomic displacement parameters (Å^2^)

**Table:**

	*U*^11^	*U*^22^	*U*^33^	*U*^12^	*U*^13^	*U*^23^
O9	0.0495 (6)	0.0571 (6)	0.0463 (5)	−0.0023 (4)	0.0031 (5)	0.0024 (4)
O8	0.0387 (5)	0.0562 (6)	0.0768 (7)	−0.0045 (4)	0.0135 (5)	−0.0033 (5)
C4	0.0394 (6)	0.0362 (6)	0.0552 (8)	0.0030 (5)	0.0080 (6)	−0.0040 (5)
C6	0.0508 (8)	0.0433 (7)	0.0488 (7)	−0.0015 (5)	0.0211 (6)	−0.0014 (5)
C2	0.0394 (6)	0.0321 (6)	0.0453 (7)	−0.0007 (4)	0.0124 (5)	−0.0025 (4)
C5	0.0390 (7)	0.0419 (7)	0.0630 (8)	−0.0004 (5)	0.0198 (6)	−0.0050 (6)
C10	0.0456 (8)	0.0586 (9)	0.0708 (10)	0.0051 (7)	−0.0008 (7)	−0.0018 (7)
C3	0.0461 (7)	0.0366 (6)	0.0427 (7)	0.0008 (5)	0.0120 (5)	0.0003 (5)
C7	0.0437 (7)	0.0411 (7)	0.0586 (8)	−0.0032 (5)	0.0175 (6)	−0.0023 (6)
C1	0.0420 (7)	0.0362 (6)	0.0429 (7)	0.0001 (5)	0.0090 (5)	−0.0019 (5)

### Geometric parameters (Å, º)

**Table:**

O9—C1	1.3589 (16)	C2—C1	1.4028 (18)
O9—H9	0.93 (3)	C5—H5	0.974 (18)
O8—C7	1.2248 (18)	C10—H10A	0.9800
C4—C5	1.398 (2)	C10—H10B	0.9800
C4—C10	1.505 (2)	C10—H10C	0.9800
C4—C3	1.3781 (19)	C10—H10D	0.9800
C6—C5	1.377 (2)	C10—H10E	0.9800
C6—C1	1.3879 (19)	C10—H10F	0.9800
C6—H6	0.989 (16)	C3—H3	0.950 (15)
C2—C3	1.4001 (18)	C7—H7	1.005 (17)
C2—C7	1.4507 (18)		
			
C1—O9—H9	104.4 (15)	H10A—C10—H10E	56.3
C5—C4—C10	120.93 (13)	H10A—C10—H10F	56.3
C3—C4—C5	117.16 (13)	H10B—C10—H10C	109.5
C3—C4—C10	121.91 (13)	H10B—C10—H10D	56.3
C5—C6—C1	119.91 (13)	H10B—C10—H10E	141.1
C5—C6—H6	121.7 (9)	H10B—C10—H10F	56.3
C1—C6—H6	118.4 (9)	H10C—C10—H10D	56.3
C3—C2—C7	120.15 (12)	H10C—C10—H10E	56.3
C3—C2—C1	119.40 (12)	H10C—C10—H10F	141.1
C1—C2—C7	120.41 (12)	H10D—C10—H10E	109.5
C4—C5—H5	118.7 (10)	H10D—C10—H10F	109.5
C6—C5—C4	122.41 (13)	H10E—C10—H10F	109.5
C6—C5—H5	118.9 (10)	C4—C3—C2	121.97 (12)
C4—C10—H10A	109.5	C4—C3—H3	120.7 (9)
C4—C10—H10B	109.5	C2—C3—H3	117.3 (9)
C4—C10—H10C	109.5	O8—C7—C2	124.41 (14)
C4—C10—H10D	109.5	O8—C7—H7	121.1 (9)
C4—C10—H10E	109.5	C2—C7—H7	114.5 (9)
C4—C10—H10F	109.5	O9—C1—C6	119.05 (12)
H10A—C10—H10B	109.5	O9—C1—C2	121.79 (12)
H10A—C10—H10C	109.5	C6—C1—C2	119.16 (12)
H10A—C10—H10D	141.1		
			
C5—C4—C3—C2	0.11 (18)	C3—C2—C1—C6	−0.87 (18)
C5—C6—C1—O9	−179.07 (12)	C7—C2—C3—C4	−177.15 (12)
C5—C6—C1—C2	0.91 (19)	C7—C2—C1—O9	−3.38 (18)
C10—C4—C5—C6	−179.24 (13)	C7—C2—C1—C6	176.64 (11)
C10—C4—C3—C2	179.27 (12)	C1—C6—C5—C4	−0.4 (2)
C3—C4—C5—C6	−0.08 (19)	C1—C2—C3—C4	0.36 (18)
C3—C2—C7—O8	−179.83 (13)	C1—C2—C7—O8	2.7 (2)
C3—C2—C1—O9	179.11 (11)		

### Hydrogen-bond geometry (Å, º)

**Table:**

*D*—H···*A*	*D*—H	H···*A*	*D*···*A*	*D*—H···*A*
C10—H10*E*···O8^i^	0.98	2.60	3.499 (2)	152
O9—H9···O8	0.94 (3)	1.77 (3)	2.6260 (17)	151 (2)
C5—H5···O8^ii^	0.974 (18)	2.607 (18)	3.4801 (18)	149.3 (13)
C6—H6···O9^iii^	0.989 (16)	2.599 (17)	3.4053 (18)	138.7 (12)

Symmetry codes: (i) *x*+1, −*y*+1/2, *z*+1/2; (ii) *x*+1, *y*, *z*; (iii) −*x*+1, −*y*+1, −*z*−1.
